# Kinetics of cellulase-free endo xylanase hyper-synthesis by *Aspergillus Niger* using wheat bran as a potential solid substrate

**DOI:** 10.1186/s12896-024-00895-w

**Published:** 2024-09-27

**Authors:** Sikander Ali, Pakeeza Noor, Muhammad Usman Ahmad, Qaiser Farid Khan, Kaynat William, Iram Liaqat, Tawaf Ali Shah, Abdulaziz Abdullah Alsahli, Youssouf Ali Younous, Mohammed Bourhia

**Affiliations:** 1 Department of Microbiology, Dr. Ikram-ul-Haq Institute of Industrial Biotechnology (IIIB), GC University Lahore, Lahore, 54000 Pakistan; 2 Depatment of Zoology, Dr. Nazir Ahmed Institute of Biological Sciences, GC University, Lahore, 54000 Pakistan; 3 College of Agriculture Engineering and Food Science, Shandong University of Technology, Zibo, 255000 China; 4 Department of Botany and Microbiology, College of Science, King Saud University, Riyadh, 11451 Saudi Arabia; 5 Evangelical College BP 1200, N’Djamena, Chad; 6 Department of Chemistry and Biochemistry, Faculty of Medicine and Pharmacy, Ibn Zohr University, Laayoune, 70000 Morocco

**Keywords:** Xylan, Optimization, Endoxylanase, *Aspergillus Niger*, Solid state fermentation

## Abstract

The present study deals with the production of cellulase-free endoxylanase by *Aspergillus niger* ISL-9 using wheat bran as a solid substrate. Endoxylanase was produced under a solid-state fermentation. Various growth parameters were optimized for the improved production of the enzyme. The Substrate level of 15 g was optimized as it provided the fungus with balanced aeration and nutrition. Among the six moisture contents investigated, Moisture Content 5 (MC5) was optimized (g/l: malt extract, 10; (NH_4_)_2_HPO_4_, 2.5; urea, 1.0) and 10 mL of MC5 was found to give the highest production of endoxylanase. The pH and time of incubation were optimized to 6.2 and 48 h respectively. The Inoculum size of 2 mL (1.4 × 10^6^ spores/mL) gave the maximum enzyme production. After optimization of these growth parameters, a significantly high endoxylanase activity of 21.87 U/g was achieved. Very negligible Carboxymethylcellulase (CMCase) activity was observed indicating the production of cellulase-free endoxylanase. The notable finding is that the endoxylanase activity was increased by 1.4-fold under optimized conditions (*p* ≤ 0.05). The overall comparison of kinetic parameters for enhanced production of endoxylanase by *A. niger* ISL-9 under Solid State Fermentation (SSF) was also studied. Different kinetic variables which included specific growth rate, product yield coefficients, volumetric rates and specific rates were observed at 48, 72 and 96 h incubation time and were compared for MC1 and MC5. Among the kinetic parameters, the most significant result was obtained with volumetric rate constant for product formation (Q_p_) that was found to be optimum (1.89 U/h) at 72 h incubation period and a high value of Q_p_ i.e.1.68 U/h was also observed at 48 h incubation period. Thus, the study demonstrates a cost-effective and environmentally sustainable process for xylanase production and exhibits scope towards successful industrial applications.

**Supplementary Information:**

The online version contains supplementary material available at 10.1186/s12896-024-00895-w.

## Introduction

 Xylan is the second most abundant polysaccharide on the earth after cellulose. It is composed of xylose molecules and is found in plant cell walls as a part of hemicellulose. It is present in annual plants, hard and soft woods in a significant quantity [10, 38]. The structural complexity of xylan demands a collective action of various enzymes to ensure complete hydrolysis of the polymer and the most important among these is β-1,4 endoxylanase [50, 62]. By nature, endoxylanase (EC 3.2.1.8) is a glycoside hydrolase which hydrolyzes β-1,4 glycosidic bonds present in the backbone of xylan and produces useful products like xylose and xylobiose as a result [26]. Over the years, endoxylanase has gathered a lot of attention due to its wide-ranging industrial applications. Perhaps one of the most widely known uses of endoxynlanase is in pulp biobleaching, as an environment-friendly alternative to carcinogenic chlorine-based chemicals [63]. Paper industries use it for drinking in reprocessing of wastepaper to lower the consumption of hazardous chemicals and to improve the paper’s brightness [15]. Endoxylanase is also utilized in the food industry for clarification of wines and fruit juices, bread making and beverage production. It is applied in the extraction of plant-derived products such as oils, starch and coffee and is used for XOS production which in turn can be used as prebiotics [29]. Additionally, endoxylanase releases nutrients and improves the digestibility of animal feedstuff. It is also known to be used in bioconversion of agro-industrial into useful products such as bioethanol [8].

Microorganisms have a high potential for endoxylanase production. Microbial sources of endoxylanase production include bacteria and fungi. Among bacteria, *B. subtilis*, *B. pumilus*, *Flavobacterium frigidium* and *Clostridium thermocellum* are the common producers. Filamentous fungi like *Aspergillus*, *Fusarium*, *Trichoderma* and *Penicillium* species are regarded as the common fungal sources of the enzyme [16]. From a commercial standpoint, filamentous fungi are preferred in comparison to bacteria as they produce xylanases in fairly high concentrations and release them extracellularly [48]. For the utilization of endoxylanase in industries, it is important for the source microorganism to be non-pathogenic and easy to culture. One such microorganism is *Aspergillus niger* which is known to have a high potential for enzyme production [22, 34]. *A. niger* is a promising candidate for endoxylanase production owing to its GRAS status, production of high enzyme yield and easy growth conditions [18]. At the industrial level, fermentative production of endoxylanase is commonly achieved using SSF and SmF. Although SSF has a longer incubation time it offers several advantages in comparison to SmF like high productivity, low production cost, reduced energy consumption, lower chances of contamination, improved stability of the enzyme, easier downstream processing and use of cheap carbon sources [9, 17, 24, 37]. In an effort to make commercial enzyme applications cost-effective, utilization of low-cost substrates like agro-wastes has been suggested [20]. Since Pakistan is an agricultural country, heaps of agricultural byproducts are produced every year which are contributing largely to environmental deterioration. Being both inexpensive and readily available these agro-wastes can be exploited as carbon sources in the fermentative production of enzymes by using microorganisms of choice. This is dually beneficial as it not only eradicates the problem of pollution caused by agricultural residues but also adds value to this lignocellulosic waste and reduces the production cost [16]. Various lignocellulosic materials like wheat bran, corncob, ragi bran, soya bran, rice husk, wheat straw, sorghum straw, sugarcane bagasse and apple pomace have been used as natural sources of xylan for endoxylanase production under SSF [23]. Through solid-state fermentation, lignocellulosic biomass can be successfully utilized for biotransformation of agricultural byproducts to improve their value and can be optimized to achieve a high yield of the target enzyme [61].

As the enzyme secreted by fungi directly depends on the culture conditions, it makes it crucial to study these and associated growth parameters to ensure the production of robust and effective enzymes. Nutritional and environmental conditions are key in enzyme production and productivity can be enhanced by determining the proper culture medium and optimum culture conditions. Various parameters like substrate concentration, substrate particle size, inoculum nature, inoculum size, time of incubation, growth temperature, aeration rate, agitation rate, nitrogen sources, volume and initial pH of moistening agent for endoxylanase production have been optimized using various isolates to maximize the overall enzyme yield [1]. Cellulase contamination reduces the efficiency of endoxylanase in the bleaching of pulp. Therefore, to make efficient use of endoxylanase in the biobleaching of pulp great attention is paid towards the production of cellulase-free endoxylanase [52]. The present study deals with the production of cellulase-free endoxylanase from *A. niger* Isl-9 using wheat bran as a solid substrate by optimizing key growth parameters.

## Materials and methods

All chemicals and reagents used in present study were of analytical grade and were obtained from the chemical store of Institute of Industrial Biotechnology (IIB), GCU Lahore.

### Bacterial strain

*A. oryzae* ISL-9 is a wild-type fungus used in current study was acquired from the culture bank of the IIB, GCU Lahore. The culture was later maintained on PDA slants for 3–5 days in a static incubator (Fischer Scientific, U.S) at 30 °C. An *Aspergillus niger*. After incubation, the slants having maximum hyphal growth and sporulation were stored at 4 °C for further use.

#### Pre-treatment of substrate

Wheat bran was acquired from agricultural farmland in the Punjab region of Pakistan and was used as a substrate. This was prepared by passing the wheat bran through a sieve (1 mm) to remove the fine powder. The sieved wheat bran was placed in sunlight for a period of 24 h to remove any moisture traces, following which; it was treated in a hot air oven at 70 °C for 30 min and stored in an air-tight container for further use.

#### Inoculum preparation

A 5-days old slant culture of *A. niger* ISL-9 was taken and 10 mL of sterile diacetyl ester of sodium sulpho succinic acid (MOT 0.05% w/v) was added aseptically into the slant culture. The fungal conidiophores were scratched gently using a sterilized inoculum loop while agitating the tube to make a homogenous suspension. Improved Neubauer Chamber was used to calculate the number of spores in the inoculum.

#### Fermentation technique

Endoxylanase was produced under SSF by *A. niger* ISL-9. Pre-treated wheat bran (10 g) was taken as the substrate in Erlenmeyer flasks of 250 mL. Ten milliliters of distilled water was used to moisten the substrate. The fermentation medium was sterilized in an autoclave (KT-40 L, ALP Ltd., Japan) at 121 °C for 15 min at 15 psi. After sterilization, the medium was allowed to cool and inoculated with 1 mL (1.4 × 10^6^ spores/mL) of the already prepared conidial suspension. The flasks were incubated at 30 °C for 72 h under static conditions.

#### Analytical techniques

After fermentation, 100 mL of sterile distilled water was poured into fermentation flasks and flasks were placed in a shaking incubator (VS-8480, Vision Scientific Co., Ltd, Korea) at 160 rpm for an hour. The crude enzyme was extracted by filtration of fermentation broth using Whatman filter paper No. 1. The filtrate obtained was stored in pre-sterilized falcon tubes and kept at 4 °C for further analysis.

##### Determination of endoxylanase activity

For enzyme activity assay, 0.5 mL of filtrate was added to 0.5 mL of 50 mM acetate buffer solution (pH 5.0) in a test tube. In this mixture 1 mL xylan (1% w/v) was added to be used as enzyme substrate and the reaction mixture was shaken thoroughly. After incubation of 30 min at 50 °C, the reaction was terminated by adding 2 mL DNS reagent into the mixture that was then incubated in a water bath (750, Daihan Scientific Co., Ltd, South Korea) at boiling temperature for 5 min. The reaction mixture was allowed to cool, and the final volume was raised to 10 mL using distilled water. The optical density was observed at 540 nm via a spectrophotometer (VIS-1100, BMS, Spain) against the blank that contained 500 µl distilled water instead of filtrate [60].

##### Enzyme activity unit

One unit of endoxylanase is defined as “the amount of enzyme that releases 1.0 µmol of reducing sugar (as xylose equivalent) per minute under the defined assay conditions”. The equation obtained from a standard curve of xylose [60]:

$$\:y=\:0.0705\text{x}+0.0107$$

$$\:x=\frac{y-0.017}{0.075}$$

Then,

1 $$Enzyme\,activity\,(U/g)=\frac{x\times{D.F}}{W}$$

Where, y = absorbance at 545 nm, x = xylose concentration released by the enzymatic hydrolysis of xylan used as substrate, D.F = dilution factor, W = weight of substrate used (g), 0.0107 and 0.0705 are the constants obtained from the xylose standard curve.

##### Determination of protein content of enzyme

The protein content of the enzyme was assayed by following the method of Bradford [6]. 5 mL of Bradford’s reagent was added to 100 µl of filtrate and mixed thoroughly. The mixture was incubated for 20 min at 30 °C. Optical density was measured at 595 nm against the control having 0.1 mL distilled water in place of filtrate.

#### Determination of cellulase activity

A reaction mixture of 1 mL was prepared by mixing 500 µl of CMC (2%) and 500 µl of filtrate. The reaction mixture was incubated at 50 °C for 30 min. The reaction was stopped by adding 3 mL of DNS reagent into the mixture and then it was incubated in a water bath at boiling temperature for 5 min. The final volume of the reaction mixture was raised to 24 mL using distilled water. The optical density was noticed at 540 nm against the blank that contained 500 µl of distilled water instead of filtrate [21].

#### Determination of turbidity of fermentation broth

The turbidity of broth obtained after filtration of the fermentation medium was determined at 595 nm using a spectrophotometer.

#### Determination of the final weight of substrate after fermentation

After fermentation, the substrate was collected and dried in sunlight for 48 h. The weight of the dried substrate was measured using an electronic weighing balance.

#### Determination of pH of fermentation broth

The pH of the fermentation broth was determined using a pH meter (PHS 3BW, BANTE instruments, U.S., Chicago).

#### Optimization of production parameters for endoxylanase production

Different parameters which included substrate (wheat bran) concentration (5 g, 10 g, 15 g, 20 g, 25 g, 30 g) moisture content (MC 1-MC 6 as mentioned in Table 1 on page 11), initial pH of medium (5.7–8.2 with intervals of 0.5), the volume of moisture content (5 mL, 10 mL, 15 mL, 20 mL, 25 mL and 30 mL), time of incubation (24 h, 48 h, 72 h, 96 h and 120 h) and inoculum size (0.5 mL – 3.0 with intervals of 0.5) were optimized to achieve the maximum production of endoxylanase. Substrate concentration was optimized by varying the range from 5 to 30 g. To optimize moisture content, 6 different moisture contents were investigated. The volume and pH of the moistening agent were optimized by varying the volume from 5 mL to 30 mL and pH from 5.7 to 8.2. The time of incubation was optimized by varying the range from 24 h to 120 h while for optimization of inoculum size, the range was varied from 0.5 to 3 mL.

**Table 1:** Effect of different moisture contents on endoxylanase activity by *A. Niger* Isl-9 under SSF

Moisture content	Composition (g/l)	pH	Fermentation technique	Microorganism	Enzyme activity (U/g)	References
MC1	Distilled water	7.8	SSF	*A. niger*	11.21 ± 0.56	Kavya and Padmavathi [30]
MC2	KNO_3_, 5; MgSO_4_, 0.1; peptone, 5; yeast extract, 5; KH_2_PO_4_,1	7	SmF	*B. pumilus*	5.72 ± 0.29	Nagar et al. [42]
MC3	Yeast extract, 2; peptone, 2; K_2_HPO_4_, 1.5; MgSO_4_, 0.5	7	SmF	*A. niger*	6.23 ± 0.31	Bakri et al. [4]
MC4	CoSO4, 0.01; CuSO4, 0.05; KH_2_PO_4_, 0.5; Yeast extract, 0.05	7	SSF	*A. niger*	1.91 ± 0.09	Park et al. [45]
MC5	Malt extract, 10; (NH_4_)_2_HPO_4_, 2.5; urea, 1	6.7	SmF	*A. niger*	13.58 ± 0.68	Pal and Khanum [43]
MC6	Xylan, 10; peptone, 1; NaNO3, 1; yeast extract, 5; KH_2_PO_4,_ 1; MgSO_4_, 0.02	5.5	SmF	*A. niger*	2.06 ± 0.103	Kalim and Ali [27]

*Incubation temperature 30 °C, wheat bran 15 g, incubation time 72 h, inoculum size 1 mL.

#### Kinetic parameters

Kinetic parameters for Solid State Fermentation (SSF) were determined following the procedures described by Lawford and Roseau [35, 49].

#### Specific growth rate

Values of specific growth rates were determined using following correlation:

2 $$\mu=\frac{dX}{h}$$

Where, µ is specific growth rate, dX is biomass while h is the time in hours.

#### Product and growth yield coefficients

The growth yield coefficient was calculated by Eq. 3.

3 $$Y_{p/x}=\frac{dp}{dX}$$

Where, Y_p/x_ is yield coefficient for product formation on the basis of biomass being developed, dp is the product being formed while dX is the amount of biomass formed.

While the product yield coefficient was calculated following equation:

4 $$Y_{p/s}=\frac{dp}{dS}$$

Where, Y_p/s_ is yield coefficient for product formation on the basis of substrate utilization, dp is the product being formed while dS is the amount of substrate consumed.

#### Volumetric rates

Volumetric rates for product formation and cellular formation were determined through following Eqs. (5 & 6):

5 $$Q_{p}=\frac{dp\times{dS}}{h}$$

6 $$Q_{x}=\frac{dp\times{dX}}{h}$$

Where, Q_p_ is volumetric rate of product formation, Q_x_ is volumetric rate for biomass formation, dp is the product being formed, dS is the amount of substrate consumed, dX is the amount of biomass formed while h is the time in hours.

#### Specific rate constants

Product formation (q_p_) and substrate utilization (q_x_) specific rate constants were determined using respective correlations (Eq. 7 and Eq. 8):

7 $$q_{p}=\mu{\times}Q_{p}$$

8 $$q_{x}=\mu{\times}Q_{x}$$

Where q_p_ is the specific rate constant for product formation, q_x_ is the specific rate constant for biomass formation, µ is specific growth rate, Q_p_ is volumetric rate of product formation while Q_x_ is volumetric rate for biomass formation.

#### Enzyme specific activity

Enzyme specific activity (E_sa_) was determined by the following equation:

9 $$E_{sa}=dP/dp$$

Where dp is the product being formed while d_P_ is the optimal product being formed.

#### Statistical analysis

Duncan’s multiple range tests were employed to determine the significance of the results [56]. The sum means values (standard deviation ± set at 5%) differ significantly from each other at *p* ≤ 0.05.

## Results and discussion

### Parametric optimizations

#### Effect of different substrate levels

**Fig. 1:**
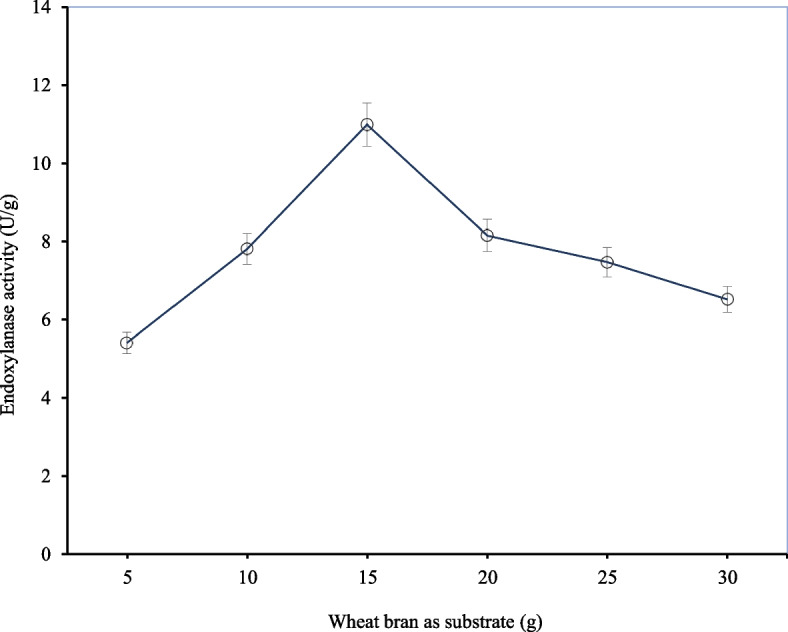
Effect of different substrate levels on endoxylanase activity by *A. niger* Isl-9 under SSF. Incubation temperature 30 °C, distilled water 10 mL, incubation period 72 h, inoculum size 1 mL

To valorize agricultural residues and develop a cost-effective method to produce the target enzyme, wheat bran was used as the substrate of choice under SSF [5, 19]. The lignocellulosic composition of wheat bran is lignin (approx. 6%), cellulose (approx. 19%), starch (approx. 19%), and non-starch polysaccharides (approx. 70%) as reported by Merali, et al. [39]. The effect of different substrate levels (5–30 g) on endoxylanase production by *A. niger* ISL-9 under SSF is shown in Fig. 1. When 5 g of pre-treated substrate was used 5.42 ± 0.27 U/g of endoxylanase activity was observed by *A. niger* ISL-9. The endoxylanase activity increased significantly by increasing the substrate level. The highest endoxylanase activity of 10.99 ± 0.55 U/g was observed at the substrate level of 15 g. By increasing the substrate level beyond 15 g, endoxylanase activity decreased gradually and significantly plummeted with increasing levels of substrate concentration due to the saturation of the enzyme’s active site. A noticeable decline in endoxylanase activity was recorded for higher levels of the substrate (25 and 30 g) (Supp. 1). The low enzyme activity at high substrate levels could be attributed to decreased aeration and enzyme deformation due to carbon catabolite repression [48]. Similarly, at a low substrate level, enzyme activity was reduced due to the insufficient nutrient supply required for encouraging the growth of *A. niger* Isl-9 [28]. The highest enzyme activity at optimal substrate level can be accredited to balanced aeration and nutrition that encouraged the growth of the fungus. Since a substrate level of 15 g gave the maximum enzyme activity, it was selected for optimization of the next parameter which was the moisture content. In a similar study, Kavya and Padmavathi [30] optimized 10 g of wheat bran for the maximum enzyme activity by using the same microorganism. In a more recent study, Morán-Aguilar and colleagues [41] found bed loading of 2 g of pretreated brewery spent grain to be optimum for xylanase production using *A. niger* CET 2700 under SSF.

#### Effect of different moisture contents

**Fig. 2:**
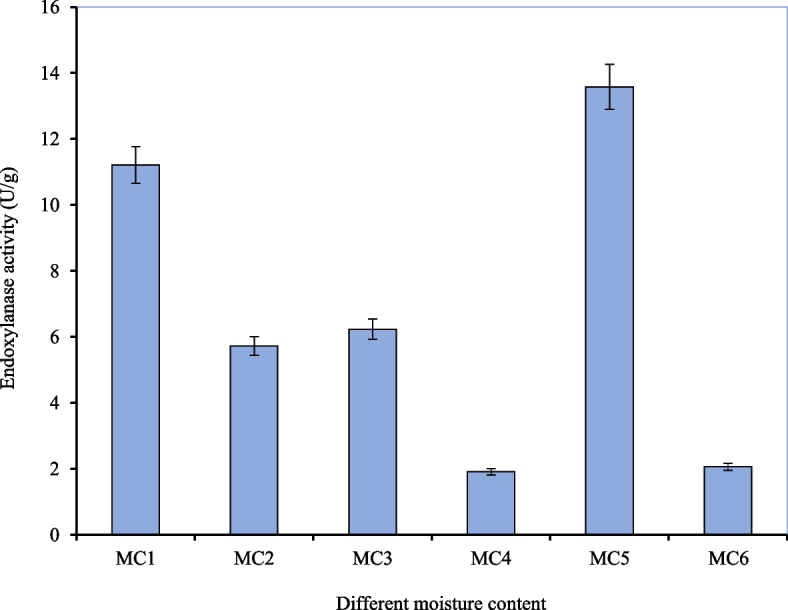
Effect of different moisture contents on endoxylanase activity by *A. niger* Isl-9 under SSF. Incubation temperature 30 °C, wheat bran 15 g, incubation time 72 h, inoculum size 1 mL

Moisture content is a critical determinant in SSF as a high level of moisture can drastically alter particle structure and porosity, on the other hand, a low moisture level can cause increased water tension and poor solubility of nutrients from the solid substrate. In the present study, the effects of six different moisture contents on enzyme production were evaluated and Fig. 2 depicts the effect of these different moisture contents on endoxylanase production by *A. niger* ISL-9 under SSF. MC1 revealed high endoxylanase activity of 11.21 ± 0.12 U/g. However, the highest endoxylanase activity of 13.58 ± 0.17 U/g was obtained with MC5 that contained (g/l): malt extract, 10; diammonium hydrogen phosphate, 2.5 and urea, 1.0. The enzyme activity assessed from the other five moisture contents under investigation was not comparable against MC5. The minimum enzyme activity (1.91 ± 0.27 U/g) was observed when MC4 was utilized. The enzyme activity obtained by using MC5 was 1.2-fold higher than that of MC1 owing to the balanced nutrition provided by MC5 required for good growth of the fungus. Also, the urea in MC5 has been reported to have a positive effect on endoxylanase production [36]. Therefore, MC5 was optimized for the parameter of the initial pH of moisture content. The effect of different moisture contents on endoxylanase activity by *A. niger* Isl-9 under SSF is shown in Table 1. Pandya and Gupte [44] optimized Mandels and Sternburg’s medium (pH 6) as the moisture content for enzyme production by *A. tubingenesis*. Desai and Iyer [13] optimized the media containing corn cob powder as a substrate for xylanase production at pH 5. Similarly. Behman et al. [7] also confirmed that moisture content and incubation time plays a significant role in the production and optimization of xylanase.

#### Effect of different initial pH of moisture content

**Fig. 3:**
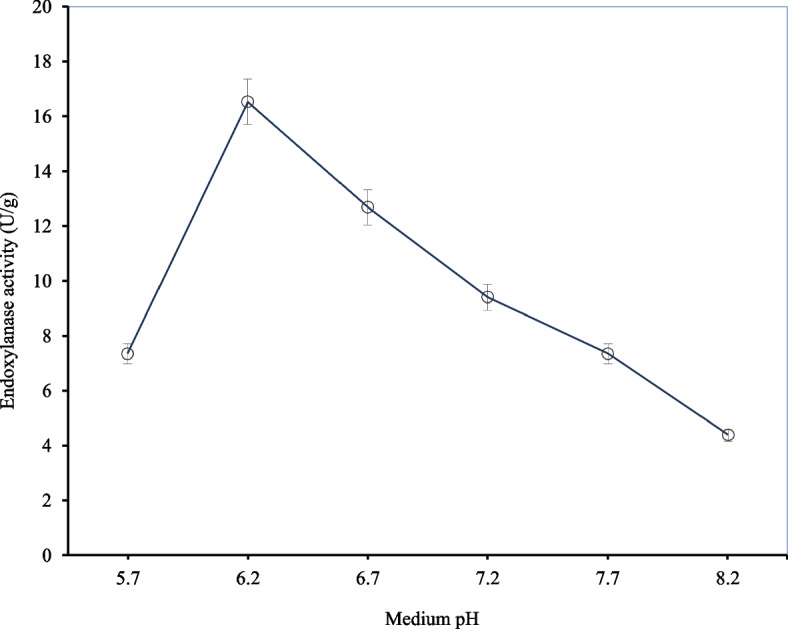
Effect of different initial pH of the moisture content on endoxylanase activity by *A. niger* Isl-9 under SSF. Incubation temperature 30 °C, wheat bran 15 g, moisture content (MC5) 10 mL, incubation time 72 h, inoculum size 1 mL

The initial pH of moisture content is crucial to the production of endoxylanase as it may directly impact nutrient availability to the producer microorganism. A range of pH values was studied Isl-9 as it directly impacts the was studied which is illustrated in Fig. 3. The pH of the moisture content varied from 5.7 to 8.2. At pH 5.7, endoxylanase activity of 7.36 ± 0.37 U/g was achieved. The highest endoxylanase activity of 16.51 ± 0.83 U/g was noticed at pH 6.2. A gradual decline in the enzyme activity was recorded by increasing the pH of moisture content beyond 6.2. The endoxylanase activity decreased significantly at a slightly alkaline pH (8.2) (Supp. 2). The highest enzyme activity at pH 6.2 may be since *A. niger* being a fungus grows well in a slightly acidic pH range of 4 to 6.5 [46] and a good fungal growth supported the maximum enzyme production at this pH. The optimum pH favoured the maximum enzyme activity as it promoted the formation of successful ES complexes by causing reversible ionization of the substrate and the enzyme. As pH 6.2 showed encouraging results for the enzyme activity, it was selected for the optimization of the volume of moisture content. In a similar study, Azzouz et al. [2] reported the highest endoxylanase activity with an initial pH of 6.0 by using the same microorganism. Michelin et al. [40] optimized the pH to 6.5 and 5.0 by using *A. terricola* and *A. ochraceus*, respectively. Sunkar et al. [57] optimized the pH to 3 for the enzyme production from *Penicillium purpurogenum*. However, Silva and Carmona [53] reported a pH of 5.5 as optimal for the enzyme production from *T. inhamatum*. Desai and Iyer [14] also reported maximum endoxylanase production at pH 5.0 for *A. niger* DX-23.

#### Effect of different volumes of moisture content

**Fig. 4:**
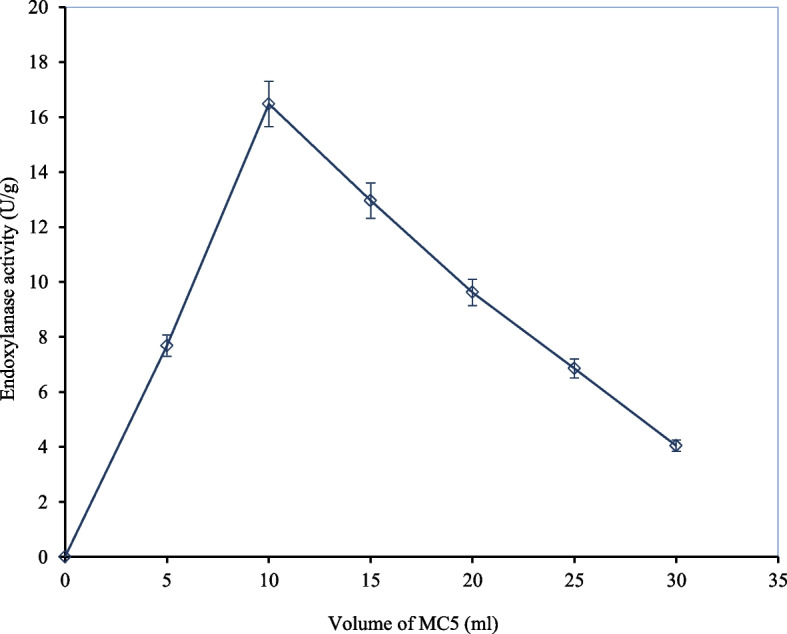
Effect of different volumes of moisture content on endoxylanase activity by *A. niger* ISL-9 under SSF. Incubation temperature 30 °C, wheat bran 15 g, pH 6.2, incubation time 72 h, inoculum size 1 mL

The volume of moisture content utilized for dampening the substrate is a critical parameter in SSF as it is directly associated with the nutrient availability, diffusion of nutrients and gaseous exchange during the fermentation process [12]. In the current study, a range of moisture levels was investigated to determine the optimum volume suited to produce the target enzyme. Figure 4 presents the effect of different volumes of moisture content (5–30 mL) on endoxylanase production by *A. niger* Isl-9 under SSF. At 5 mL of moisture level, endoxylanase activity of 7.68 ± 0.38 U/g was achieved and this activity kept increasing with the increase in moisture level. The highest endoxylanase activity of 16.48 ± 0.82 U/g was obtained with 10 mL of moisture content. Any further increase in the volume of moisture content beyond 10 mL, led to a gradual decline in the enzyme activity up a drastic decline in enzyme activity at 30 mL of moisture content was observed which can be linked to the retardation of growth of the producer microorganism due to decreased oxygen supply because of generation of thick water film (Supp. 3). It was noted that the addition of moisture level beyond what could be easily absorbed by the substrate negatively impacted enzyme production by impacting the particle size and decreasing the ease of gaseous exchange. Similarly, at low moisture levels, enzyme production was reduced as there was insufficient nutrient supply due to reduced nutrient solubility in the solid substrate as reported by Ikasari and Mitchell [25]. Thus, moisture content significantly affected the productivity under SSF as its optimal level encouraged the microbe to efficiently utilize the substrate. The optimization of the next parameter (time of incubation) was carried out with 10 mL of moisture content as the optimum enzyme activity was noticed at this volume. Pandya and Gupte [44] reported moisture level at 1:5 as optimal for enzyme production by *A. tubingensis*. However, Tai et al. [58] reported the maximum xylanase activity with 60% moisture content by using the same microorganism. In another study, Kheng and his coworkers [31] reported a 43% moisture level as optimum for endoxylanase production by *A. niger* utilizing Palm Kernel Cake as a solid substrate. Morán-Aguilar et al., [41] reported 80% moisture content to be optimum to produce xylanase using *A. niger* CECT 2700 by using brewery spent grain in SSF. Interaction between factors such as incubation time, moisture content, temperature, pH, volume of substrate plays a significant part in production of enzyme [54].

### Effect of time-dependent incubation

**Fig. 5:**
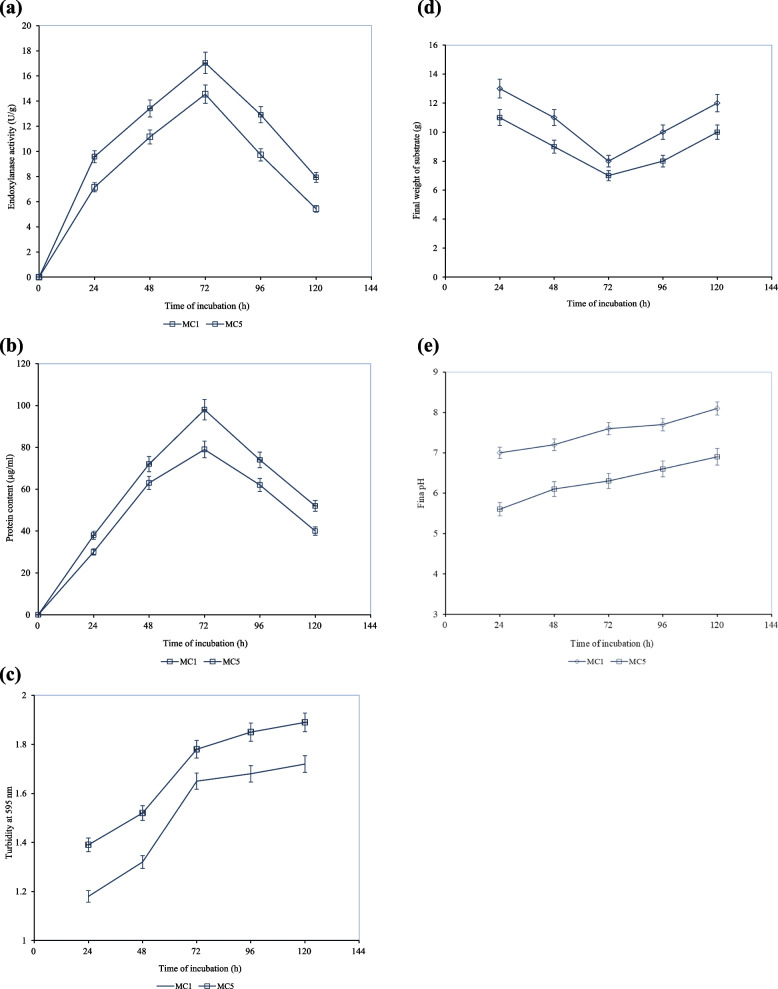
**a** Effect of different times of incubation on endoxylanase activity by *A. niger* Isl-9 under SSF. **b** Effect of different times of incubation on protein content of enzyme by *A. niger* Isl-9 under SSF. **c** Effect of different times of incubation on the turbidity of fermentation broth by *A. niger* Isl-9 under SSF. **d** Effect of different times of incubation on the final weight of the substrate by *A. niger* Isl-9 under SSF. **e** Effect of different times of incubation on final pH of fermentation broth by *A. niger* Isl-9 under SSF. Incubation temperature 30 °C, wheat bran 15 g, MC1 10 mL at pH 7.8, MC5 10 mL at pH 6.2, inoculum size 1 mL)

 The effect of time of incubation is a key parameter that can impact the overall endoxylanase production by *A. niger* Isl-9 under SSF (Supp. 4). The time of incubation varied in a range between 24 and 120 h. Since MC1 and MC5 showed comparatively high values of enzyme activity, the effect of time of incubation was compared for MC1 and MC5 separately. An endoxylanase activity of 9.58 ± 0.48 U/g was recorded at the incubation time of 24 h. High endoxylanase activity was observed with an increase in incubation temperature. The maximum endoxylanase activity of 17.04 ± 0.85 U/g was achieved at an incubation time of 72 h (Fig. 5a). Different parameters (viz. protein content, turbidity, weight of substrate and pH) were studied to thoroughly assess the suitability of the temperature of incubation The protein content at incubation time of 72 h was found to be 98 µg/mL while turbidity was 1.78. The weight of the substrate was reduced from 15 to 7 g while pH was increased from 6.2 to 6.3 after an incubation period of 72 h.

The effect of incubation time on protein content, turbidity, weight of substrate and pH is shown in Fig. 5b-e, respectively. By increasing the incubation time beyond 72 h, a gradual decline in the enzyme activity was recorded and the enzyme activity decreased significantly at incubation time of 120 h. The highest enzyme activity was achieved at the optimum time of incubation due to exponential growth and active production of primary metabolites (endoxylanase) by the microorganism. Kar et al., [28] also reported a substantial decrease in xylanase production when temperature was increased beyond the optimum for *Trichoderma reesei* SAF3 utilizing wheat bran as substrate in SSF. Kumari et al. [33] optimized *Bacillus australimaris* KS2 at 24 h incubation supplemented with 2.0% wheat bran to produce hyper xylanase. However, maximum production (588 mg/g) in terms of enzymatically hydrolyzed substrate was achieved at 16 h at pH 6. In recent findings [11] achieved optimal xylanase production by *A. Niveus* after 8 h of incubation.

As the time of incubation was prolonged, the stationary phase of microbial growth prevailed, and nutrient depletion and proteolytic degradation of the enzyme at the late stationary phase resulted in the degradation of enzyme activity. Poor xylanase activity beyond 72 h is likely associated with the production of reducing sugars like xylose, glucose and fructose in the culture medium which may subsequently repress endoxylanase production Moreover, the prominent decrease in enzyme production can be deemed as a joint result of the reduction in nutrients of the medium and catabolic repression of the enzyme. With further increase in incubation time, the decline phase started which significantly lowered the enzyme activity. MC5 gave 1.1-fold higher enzyme activity than that of MC1. As incubation times of 48 and 72 h exhibited encouraging results for the enzyme activity, optimization of inoculum size was carried out with both incubation times using MC5 as moisture content. Like the current study, Perez-Rodriguez et al. [47] also reported maximum enzyme activity with an incubation time of 72 h (3 days) by using the same producer microorganism. Similarly, an incubation period of 144 h (6 days) was optimized for xylanase production by *A. niger* NFCCI 4113 using wheat bran as a solid substrate by Kumar et al. [32]. Contrastingly, another study reports maximum xylanase production following 7 days of incubation by *A. niger* under SSF [51].

### Effect of different inoculum sizes on endoxylanase activity

**Fig. 6:**
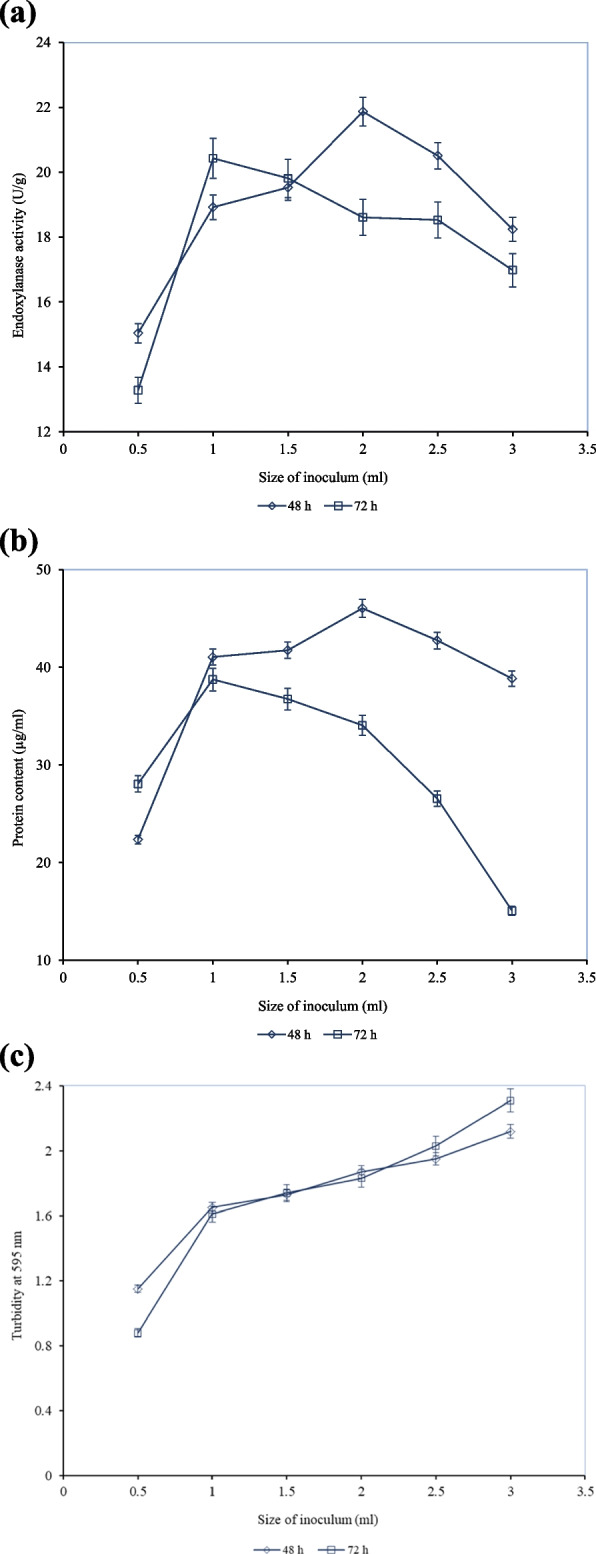
**a** Effect of different inoculum size on endoxylanase activity by *A. niger* Isl-9 under SSF. **b** Effect of different inoculum sizes on the protein content of the enzyme by *A. niger* Isl-9 under SSF. **c** Effect of different inoculum sizes on the turbidity of fermentation broth by *A. niger* Isl-9 under SSF. Incubation temperature 30 °C, wheat bran 15 g, moisture content 10 mL, pH 6.2, incubation time 48 h,72 h

The Fig. 6a shows the effect of different inoculum sizes (0.5-3 mL) on endoxylanase production by *A. niger* Isl-9 under SSF. The effect of the size of inoculum was compared for 48 and 72 h as they both showed relatively high endoxylanase activity. When the inoculum size of 0.5 mL was used, endoxylanase activity of 15.041 ± 0.75 U/g was achieved. The enzyme activity was increased by increasing the inoculum size. The highest endoxylanase activity of 21.871 ± 1.09 U/g was observed with the inoculum size of 2 mL. Azzouz Zahra et al. [3] also reported optimal conditions for xylanase production in which inoculum size of 1.91.9 × 10⁷spores/mL was used.

At an inoculum size of 2 mL, protein content was found to be 46.05 µg/mL while turbidity was recorded to be 1.871 as shown in Fig. 6b and c respectively. The enzyme activity declined gradually with the increase in inoculum size beyond the optimal. The endoxylanase activity achieved after 48 h of fermentation with an inoculum size of 2 mL (8%) was 0.85-fold higher than the enzyme activity obtained with an inoculum size of 1 mL (4%) after 72 h of fermentation (Supp. 5). The low enzyme activity at lower inoculum size may be explained in terms of the insufficient number of microbial cells required for efficient substrate utilization and enzyme production. The decreased enzyme activity at high inoculum size may be due to nutrient depletion by the increasing biomass which diminishes metabolic activity. However, the present study contradicts Siti et al. [54] findings in which inoculum size showed insignificant effect towards enzyme activity.

Optimal inoculum size maintained a balance between biomass synthesis and accessible nutrients therefore it gave the maximum enzyme activity [55]. Finally, an incubation time of 48 h and inoculum size of 2 mL was optimized for endoxylanase production by *A. niger* Isl-9 under SSF. Kavya and Padmavathi [30] reported the maximum enzyme activity with an inoculum size of 10^−1^ dilution by using the same microorganism. In related studies of xylanase production, Selim et al., [51] reported the spore suspension of 2 × 10^5^ as optimum for *A. niger* whereas Sunkar et al. [57] optimized the inoculum size of 1.2 × 10 [7] spores/mL by using *P. purpurogenum*. Vu, V. et al. [59] used *Bacillus* strains and their consortium to degrade wheat bran substrate and reported increased xylanase production when *Bacillus* strains were used in consortium.

## Comparison of kinetic parameters

The overall comparison of kinetic parameters for enhanced production of endoxylanase by *A. niger* Isl-9 under SSF is shown in Table 2. Different kinetic variables were studied. These variables were observed at 48, 72 and 96 h incubation time and were compared for MC1 and MC5. The maximum specific growth rate (µ) of 0.032 g/h was observed at 48 h incubation period. The yield coefficient for product formation based on dry cell weight formation (Yp/x) was found to be the highest (9.57 U/g/g) at an incubation period of 72 h while the yield coefficient for product formation based on substrate consumption (Yp/s) was maximum (2.24 U/g/g) at 48 h incubation period. The highest volumetric rate constant for dry cell weight formation (Qx) of 0.2 g/g/h was obtained at an incubation time of 72 h. The specific rate constant for product formation (qp) and specific rate constant for dry cell weight formation (qx) was found to be maximum at the 48 h incubation period. The maximum specific enzyme activity of 0.19 U/g was noticed at the 48 h incubation period. Qp is the most significant kinetic parameter that indicates the net productivity of the enzyme. The higher the Qp value, the higher the enzyme activity and productivity would be. The highest volumetric rate for production formation (Qp) of 1.89 U/h was achieved at 72 h incubation time and at the 48 h incubation period, QP value was found to be high (1.68 U/h). The results obtained from the comparison of kinetic parameters (*p* ≤ 0.05) were found to be closely related to the results obtained under optimized conditions (*p* ≤ 0.05) which indicates the statistical significance of the results. A similar trend was observed by various researchers Lawford and Roseau [35, 49]. de Oliveira Simas, A.L. [11] achieved maximum xylanase (4.66 ± 1.38 U/mg) production by *Aspergillus niveus* using wheat bran as substrate.

**Table 2:** Overall comparison of kinetic parameters for enhanced endoxylanase production by *A. Niger* Isl-9 under SSF

Kinetic variables	Kinetic quotients	units	Kinetic models	Kinetic modes	Time of incubation					
					48 h		72 h		96 h	
					MC1	MC5	MC1	MC5	MC1	MC5
Specific growth	µ	g/h	Growth	Cellular formation	0.028 ± 0.0014	0.032 ± 0.0016	0.023 ± 0.00115	0.025 ± 0.00125	0.018 ± 0.0009	0.019 ± 0.00095
Product yield	Y_P/X_	U/g/g	Coefficient	Metabolic production	8.45 ± 0.4225	8.83 ± 0.4415	8.82 ± 0.441	9.57 ± 0.4785	5.79 ± 0.2895	6.98 ± 0.349
Product yield	Y_p/s_	U/g/g	Coefficient	Metabolic production	2.79 ± 0.1395	2.24 ± 0.112	2.08 ± 0.104	2.13 ± 0.1065	1.95 ± 0.0975	1.85 ± 0.0925
Volumetric rate	Q_p_	U/h	Constant	Endoxylanase production	0.93 ± 0.0465	1.68 ± 0.084	1.41 ± 0.0705	1.41 ± 0.0255	0.51 ± 0.0255	0.94 ± 0.047
Volumetric rate	Q_x_	g/g/h	Constant	Cellular formation	0.11 ± 0.0055	0.19 ± 0.0095^a^	0.16 ± 0.008	0.16 ± 0.0045	0.09 ± 0.0045	0.13 ± 0.0065
Specific rate	Q_p_	U/g/h	Rate	Endoxylanase production	0.026 ± 0.0013	0.054 ± 0.0027	0.032 ± 0.0016	0.032 ± 0.00045	0.009 ± 0.00045	0.017 ± 0.0085
Specific rate	q_x_	g/h/h	Rate	Cellular formation	0.003 ± 0.0015	0.006 ± 0.0003	0.004 ± 0.0002	0.004 ± 0.0001	0.002 ± 0.0001	0.0024 ± 0.00012
Specific enzyme activity	Esa	U/g	Activity	Protein formation	0.18 ± 0.009	0.19 ± 0.0095	0.18 ± 0.009	0.18 ± 0.008	0.16 ± 0.008	0.17 ± 0.0085

^a^Incubation temperature 30 °C, wheat bran 15 g, moisture content 10 mL, pH 6.2

Kinetic parameters: Yield coefficient for product formation on the basis of dry cell weight formation = Y_p/x_ (U/g/g), Yield coefficient for product formation on the basis of substrate consumption = Y_p/x_ (U/g/g), Volumetric rate constant for product formation = Q_p_ (U/h), Volumetric rate constant for dry cell weight formation = Q_x_ (g/g/h), Specific rate constant for product formation = q_p_ (U/g/h), Specific rate constant for dry cell weight formation = q_x_ (g/h/h) and Specific enzyme activity on the basis of protein formation = ESA (U/g).

## Conclusion

In the present study, endoxylanase enzyme was produced from *Aspergillus niger*. This enzyme was produced by optimizing the various growth parameters under SSF using wheat bran as substrate. Among the parameters studied, the incubation period and inoculum size significantly affected the production of endoxylanase. After optimizing various growth parameters, endoxylanase activity of 21.87 U/g was observed. The overall enzyme activity was increased by 1.4-fold under optimized conditions (*p* ≤ 0.05). Among the kinetic parameters, the volumetric rate for product formation (Qp) gave the most notable result (1.89 U/h) at an incubation period of 72 h. Very negligible CMCase activity was detected which clearly indicated that the enzyme produced under optimized conditions was cellulase free. However, scale-up studies are required before the commercial utility of the enzyme.

## Supplementary Information

Supplementary Material 1.

## Data Availability

Data will be available upon request from the corresponding author.
