# Supplementary material for: Kinetics of cellulase-free endo xylanase hyper-synthesis by Aspergillus Niger using wheat bran as a potential solid substrate
Source: BMC Biotechnol. 2024 Sep 27;24:69. doi: 10.1186/s12896-024-00895-w (PMC11438087; doi:10.1186/s12896-024-00895-w)
Supplement: Supplementary file 1 — Supplementary Material 1. [file 12896_2024_895_MOESM1_ESM.docx]

**Kinetics of cellulase-free endoxylanase hyper-synthesis by *Aspergillus niger* using wheat bran as a potential solid substrate**

**Supplementary material**

**Supplementary Table. 1:** Effect of different substrate levels on endoxylanase activity by *A. niger* Isl-9 under SSF.

| Wheat bran (g) | Endoxylanase activity (U/g) |
| --- | --- |
| 5 | 5.42± 0.27 |
| 10 | 7.82±0.39 |
| 15 | 10.99±0.55 |
| 20 | 8.16±0.41 |
| 25 | 7.48±0.37 |
| 30 | 6.53±0.33 |

Incubation temperature 30°C, distilled water 10 ml, incubation period 72 h, inoculum size 1 ml.

Standard deviations (± set at 5%) amongst the values of three parallel replicates are indicated by ±. The sum means values significantly differ from each other at p≤ 0.05.

**Supplementary Table. 2:** Effect of different initial pH of moisture content on endoxylanase activity by *A. niger* Isl-9 under SSF.

| pH | Endoxylanase activity (U/g) |
| --- | --- |
| 5.7 | 7.36±0.37 |
| 6.2 | 16.51±0.83 |
| 6.7 | 12.68±0.63 |
| 7.2 | 9.41±0.47 |
| 7.7 | 7.36±0.37 |
| 8.2 | 4.4±0.22 |

Incubation temperature 30°C, wheat bran 15 g, moisture content (MC5) 10 ml, incubation time 72 h, inoculum size 1 ml.

Standard deviations (± set at 5%) amongst the values of three parallel replicates are indicated by ±. The sum means values significantly differ from each other at p≤ 0.05.

**Supplementary Table. 3:** Effect of different volumes of moisture content on endoxylanase activity by *A. niger* Isl-9 under SSF

Incubation temperature 30°C, wheat bran 15 g, pH 6.2, incubation time 72 h, inoculum size 1 ml.

Standard deviations (± set at 5%) amongst the values of three parallel replicates are indicated by ±. The sum means values significantly differ from each other at p≤ 0.05.

**Supplementary Table. 4:** Effect of different times of incubation on endoxylanase activity, total protein content, broth turbidity, final weight of substrate and final pH of fermentation broth by *A. niger* Isl-9 under SSF.

Incubation temperature 30°C, wheat bran 15g, MC1 10ml at pH 7.8, MC5 10ml at pH 6.2, inoculum size 1ml.

Standard deviations (± set at 5%) amongst the values of three parallel replicates are indicated by ±. The sum means values significantly differ from each other at p≤ 0.05.

**Supplementary Table. 5:** Effect of different inoculum size on endoxylanase activity, total protein content and turbidity of fermentation broth by *A. niger* Isl-9 under SSF.

| \| Volume (ml) \| % (v/w) \| 48 h \| 72 h \| 48 h \| 72 h \| 48 h \| 72 h \| \| --- \| --- \| --- \| --- \| --- \| --- \| --- \| --- \| \| 0.5 \| 2±0.1 \| 15.04±0.75 \| 13.28±0.66 \| 22.34±1.12 \| 28.05±1.40 \| 1.15±0.06 \| 0.88±0.04 \| \| 1 \| 4±0.2 \| 18.92±0.95 \| 20.43±1.02 \| 41.05±2.05 \| 38.75±1.94 \| 1.65±0.08 \| 1.61±0.08 \| \| 1.5 \| 6±0.3 \| 19.53±0.98 \| 19.81±0.99 \| 41.75±2.08 \| 36.75±1.84 \| 1.73±0.09 \| 1.74±0.09 \| \| 2 \| 8±0.4 \| 21.87±1.09 \| 18.61±0.93 \| 46.05±2.3 \| 34.05±1.7 \| 1.87±0.093 \| 1.83±0.1 \| \| 2.5 \| 10±0.5 \| 20.51±1.02 \| 18.53±0.92 \| 42.75±2.14 \| 26.55±1.33 \| 1.95±0.1 \| 2.03±0.101 \| \| 3 \| 12±0.6 \| 18.24±0.91 \| 16.98±0.85 \| 38.85±1.94 \| 15.05±0.75 \| 2.12±0.106 \| 2.301±0.12 \| |
| --- | --- | --- | --- | --- | --- | --- | --- | --- | --- | --- | --- | --- | --- | --- | --- | --- | --- | --- | --- | --- | --- | --- | --- | --- | --- | --- | --- | --- | --- | --- | --- | --- | --- | --- | --- | --- | --- | --- | --- | --- | --- | --- | --- | --- | --- | --- | --- | --- | --- | --- | --- | --- | --- | --- | --- | --- |

Incubation temperature 30°C, wheat bran 15 g, moisture content 10 ml, pH 6.2, incubation time 48 h, 72 h.

Standard deviations (± set at 5%) amongst the values of three parallel replicates are indicated by ±. The sum means values significantly differ from each other at p≤ 0.05.
